# Moving forward to address key unanswered questions on targeting PD-1/PD-L1 in cancer: limitations in preclinical models and the need to incorporate human modifying factors

**DOI:** 10.1186/s40425-019-0789-4

**Published:** 2019-11-07

**Authors:** Catherine T. Le, William J. Murphy

**Affiliations:** 1 Department of Dermatology, School of Medicine, University of California, Davis, Sacramento, CA USA; 2 Department of Internal Medicine, School of Medicine, University of California, Davis, Sacramento, CA USA

**Keywords:** PD-1, PD-L1, Exhaustion, Priming, T-cell, Preclinical modeling, Immune checkpoint inhibitors

## Abstract

The tremendous clinical success of immune checkpoint inhibition (ICI), particularly targeting the programmed cell death protein 1 (PD-1)/programmed death-ligand 1/2 (PD-L1/2) pathway, has resulted in application to multiple cancers, as a monotherapy and as a companion to both conventional and novel agents. Despite this, the precise mechanisms underlying the anti-tumor effects of PD-1/PD-L1 blockade remain unclear. Emphasis has centered on its reversal of tumor-specific CD8+ T-cell exhaustion, although many cell types and processes are likely impacted. Due to the complex and pervasive roles of PD-1/PD-L1 on T-cell biology, including on initial T-cell priming, PD-1 blockade likely affects all aspects of T- cell responses, and these other effects may be even more critical for durable anti-tumor responses. Delineating these complex interactions necessitates in vivo modeling. By far, the healthy, young and inbred laboratory mouse, transplanted with an extensively cultured tumor cell line, has been the predominant preclinical model used to assess potential therapeutic efficacies. However, these mouse models often do not adequately reflect the tumor progression and cellular and genetic heterogeneity found within human cancers. Furthermore, laboratory mice also present with a vastly restricted immune profile compared to humans. This commentary discusses some of the critical questions that need to be addressed to optimize the use of ICI as well as caveats and limitations for consideration when extrapolating preclinical mouse data to the human cancer scenario.

The recent successes and improved safety profiles of ICI in cancer therapy, particularly targeting programmed cell death protein 1 (PD-1) and its ligands (PD-L1/2), have resulted in approval for several solid and hematologic malignancies, even as first-line therapy [1]. Other promising ICI in development include blockade therapy against T-cell immunoglobulin and mucin-domain containing-3 (TIM3), Lymphocyte-activation gene 3 (LAG3), and T-cell immunoreceptor with Ig and ITIM domains (TIGIT). In addition, combination approaches targeting both cytotoxic T-lymphocyte-associated protein 4 (CTLA-4) and PD-1/PD-L1 are being assessed clinically. As adoptive T-cell therapies, including chimeric antigen receptor (CAR) T-cell therapies, are being used increasingly, efforts have been directed to augment persistence and function of tumor-specific T cells [2]. Clinical success has generated increased attention regarding mechanisms of action. Such insights could optimize and shift therapeutic application for greater efficacy and outcome as well as reduce potential toxicities. Critical questions remain on how, when, and to whom ICI should be applied and on potential effects, both positive and negative, when combined with other modalities. Questions also remain on what the proper duration of ICI therapy is and, if therapy is discontinued, how long are the effects of ICI therapy maintained. This is especially pertinent given the property of many cancers to become dormant and evade immune attack and the well-reported decline in immune function with age. All of which could mean that cessation of immunotherapy, even in cases of complete responses, could be viewed as a potential risk for relapse. Finally, understanding the long-term impact of ICI on the overall immune status is critical, especially in older individuals, who have a finite memory T-cell pool. Preclinical mouse models are invaluable tools that can address some of these pressing questions. However, aside from inherent species differences and the difficulty of assessing immune effects using xenogeneic models, other critical caveats need to also be considered. Understanding the advantages and disadvantages inherent to mouse preclinical tumor modeling is, therefore, paramount in moving forward with PD-1/PD-L1 targeting in cancer therapy.

## Diverse role of PD-1/PD-L1 signaling on T cells

The initial goal of PD-1/PD-L1 targeting in cancer centered on reinvigorating tumor-specific but functionally exhausted memory CD8+ T cells. T-cell exhaustion, originally described in chronic viral infection models, denotes a state of chronic antigen exposure that impairs the transition from effector to memory state. Exhaustion is an umbrella term that demarcates specific properties: co-expression of one or more inhibitory receptors (i.e. PD-1, TIM3, LAG3), reduced proliferative capability, and decreased cytokine production (tumor necrosis factor, interferon-gamma) and effector functions [3]. T-cell exhaustion can result from chronic antigen stimulation but may also be induced by other immunosuppressive pathways, such as inflammatory tissue microenvironment, presence of regulatory immune cell populations, and other inhibitory signals from cytokines and receptors [3]. The expression of PD-1 itself is not solely a marker of exhaustion, as PD-1 is also rapidly upregulated by naïve T cells upon initial activation [1, 3]. Therefore, determination of T-cell exhaustion necessitates the assessment of functional readouts. PD-1 has been demonstrated to decrease CD28 co-stimulatory signaling, which reinforces the concept that PD-1 is involved in both initial naïve T-cell priming and memory T-cell exhaustion [4]. Initial work on the role of PD-1 in T-cell exhaustion were performed using viral response models. Studies utilizing chronic lymphocytic choriomeningitis virus (LCMV) infection in mice highlighted the ability of PD-L1 blockade to reverse exhaustion of LCMV-specific CD8+ T-cells [5]. However, PD-1 knockout mice infected with chronic LCMV surprisingly had greater accumulation of dysfunctional T cells and decreased memory maintenance suggesting that PD-1 also has a potentially important role in allowing memory T cells to persist in the presence of chronic antigen stimulation [6]. Some mouse models employ transient CD4 depletion to augment the exhausted phenotype in the CD8+ T-cell pool [5, 6]. However, CD4+ T cell depletion has been demonstrated to negatively impact CD8+ T-cell priming in a phenomenon termed “helplessness,” and while “helplessness” may result in many of the same characteristics as “exhaustion,” it remains unclear what the commonalities are in these two pathways. Considering the diverse functions of the PD-1/PD-L1 pathway on T-cell biology, it is not known if blockade is effective primarily by reversing T-cell exhaustion on memory T cells or augmenting priming and epitope-spreading of naïve T cells. Promoting priming may allow for continuous adaptation to cancer, which is genetically unstable and immune evading. Likely, both processes are contributing to the long-term successes of ICI in some patients but may be dependent on both the timing of the therapy and the genetic stability of the cancer.

Thus, given the ever-increasing application of ICI to PD-1/PD-L1 in many cancer regimens, even as front-line therapy, it is imperative to more thoroughly understand the precise mechanisms of action. Preclinical mouse models in immunology predominantly use blocking or depleting monoclonal antibodies or genetic deletion to ascertain function of a molecule or pathway. Limitations of these approaches arise due to incompleteness of blockade, unintended effects on other cell types, unpredicted compensatory pathways, or negative effects on normal development or immune cell differentiation. Effective use of monoclonal antibodies or small molecules is also dependent on pharmacokinetics, optimal exposure, and inherent antagonistic versus agonistic properties, which may not be mutually exclusive. Another important caveat in preclinical mouse cancer immunotherapy models is related to the lack of “murinized” reagents analogous to humanized reagents used in patients. Preclinical models often necessitate the use of xenogeneic antibodies, which will elicit a range of immune responses, including neutralizing antibodies and, in some cases, fatal anaphylaxis with repeated application of rat or hamster monoclonal antibodies to mouse PD-1 [7]. As the impact of long-term immunotherapy in most preclinical tumor models cannot be determined with xenogeneic reagents, the ability to use mouse reagents becomes more important to model the potential long-term effects of ICI. One way to possibly model human ICI reagents in vivo is the utilization of “humanized” mice, either created through the reconstitution of immunodeficient mice with human cells or “humanization” of specific checkpoints in immunocompetent mice [8]. However, the xenogeneic environment has profound effects on human immune cell development, engraftment and function, which can confound interpretation of the data when modeling primary human immune responses to weakly immunogenic, autologous tumors or worse yet, allogeneic human tumor lines.

Another uncertainty surrounding the mechanism of PD-1/PD-L1 blockade concerns the widespread expression of these molecules on various immune cells as well the cancer itself. While PD-1 has been extensively described on T cells, there are reports of PD-1 on natural killer (NK) cells, B cells, and monocytes [1]. Indeed, studies using PD-1 knockout mice detail abnormalities in B cell function and neutrophil response [9, 10]. Caution must be exercised when assessing PD-1/PD-L1 expression solely by flow cytometry. It has been recently reported that non-specific binding of PD-1 antibodies by dead or dying cells can occur, leading to possible false-positive results [11]. Furthermore, PD-L1 can be ubiquitously expressed by all cells under inflammatory and activating conditions [1]. It, therefore, remains unclear with PD-1/PD-L1 blockade if the resulting or indirectly/or indirectly on T cells.

## Limitations of preclinical modeling in reflecting human cancer progression and host factors

The overwhelming majority of preclinical tumor studies utilize fully transformed, extensively cultured, rapidly growing (growth fraction is usually 100%), and relatively homogeneous tumor cell lines. These tumor cell lines are typically engrafted into healthy, young (usually 8–10 weeks old, analogous to a young human adolescent), genetically identical, and inbred laboratory mice. Because research mice are housed under strict specific-pathogen-free (SPF) conditions, even a “middle-aged” mouse presents as immunologically naïve and immature, especially when compared to mice housed under “dirty” conditions [12]. The naïve immune system can readily respond to undefined and highly immunogenic determinants on tumor cell lines, particularly when injected into subcutaneous tissues which result in tissue damage and subsequent toll receptor triggering. Though tumor lines used are considered syngeneic, immunogenicity still is evident and unpredictable due to extensive culturing. Differences between tumor cell lines and mouse strains are highlighted even more so when considering variations between vendors due to genetic drift. Tumor lines that are transformed to express viral or xenogeneic antigens, such as ovalbumin (OVA), to monitor putative “tumor-specific” T-cells elicit strong primary and potentially artefactual T-cell responses. The tumor cell lines, due to extensive in vitro passaging under confluent conditions, are homogeneous and undergo massive cell death during in vivo engraftment. This is reflected in the tumor growth kinetics, where a lag period of a week or two is followed by extremely rapid growth. When the tumors are implanted subcutaneously, clinical survival is due to primary tumor size or necrosis and rarely metastasis. Immune resistance to tumor growth, therefore, represents an acute response to engraftment. This suggests that ICI efficacy in preclinical models may be due to an augmentation of a primary response rather than reversal of T-cell exhaustion since immune evasion by the tumor would not be necessary until later. This is in stark contrast to the chronic viral models, which can take over 6 weeks to establish T-cell exhaustion following infection. Thus, the “exhausted” phenotype observed during this acute primary response to a tumor line is unlikely to reflect the “exhausted” phenotype observed in human patients, where cancer may have been progressing for years, is vastly heterogeneous, and employs numerous immune evasion mechanisms.

**Fig. 1:**
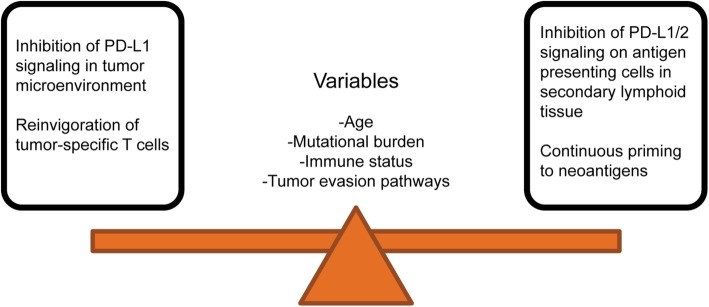
Mechanisms of PD-1/PD-L1 blockade in human cancer. The main mechanisms by which PD-1 blockade mediates anti-tumor effect, either reinvigoration of tumor-specific T cells in the microenvironment or promoting continuous priming of neoantigens, are balanced by modifying factors: age, mutational burden, immune status, and tumor evasion pathways

In preclinical models, ICI treatment is sometimes initiated at the time or soon after the tumor injection, such that the animal’s tumor burden is minimal compared to the human cancer scenario. The initiation of ICI in human cancer patients begins in a setting where immune evasion has already occurred and where both priming and exhaustion of T cells are possibly simultaneously occurring (Fig. 1). Modeling slower growing or spontaneous tumors, such as in genetically engineered mouse (GEM) models, would better mirror the human cancer scenario but are difficult to use in therapeutic intervention studies due to heterogeneity in tumor growth. This necessitates large sample sizes and higher cost. However, in the end, such approaches may yield more robust and meaningful data for our understanding of the most effective application of cancer immunotherapy regimens in humans.

While the predominant emphasis in cancer therapy has centered on the cancer, a key component integral for immunotherapy efficacy is the immune status of the patient with age being a driving factor. Given that the median age of cancer diagnosis is 66 years of age, according to NCI’s Surveillance, Epidemiology, and End Results Program, the differences in the T-cell memory compartment are stark when comparing young (8–10 weeks) to aged (> 16 months) inbred mice housed under SPF conditions. Likewise, the human immune system is dynamic and changes with exposure to multiple infections, environmental factors, age, thymic involution, and other host factors; yet, these elements are often not modeled but likely influence ICI response and tumor growth. As both the memory T-cell compartment and PD-1/PD-L1 expression increases with age and with presence of chronic or latent viral infection, such as CMV and EBV, the fact that the majority of mouse models do not mirror these conditions is a concern when attempting to extrapolate immune therapy effects to the clinical scenario [13].

In addition, other human modifying factors such as diet, sex, age, gut microbiome, co-morbidities and adiposity can influence immune responses and cancer immunotherapy outcome [14–17]. We have observed that obesity has a profound impact on T-cell phenotype and function in mice, dogs, non-human primates, and humans [15]. Furthermore, although obesity promoted PD-1-mediated T-cell exhaustion as well as tumor progression, it also paradoxically promoted anti-tumor responses to PD-1 blockade in mice and was associated with increased progression-free survival clinically [15]. Other clinical studies have reported an impact of gender on outcome as well, highlighting the critical importance of incorporating human modifying factors in preclinical models [14]. However, like GEM models, the incorporation of human modifying factors, such as age, sex, or obesity, adds tremendous time and cost. Although initial preclinical studies with young, inbred SPF mice are cost-effective to determine early dosing and timing and to delineate efficacy versus toxicities, these models are simply not sufficient to directly extrapolate with regard to efficacy. This is particularly pertinent given the off-target effects and potentially life-threatening toxicities that have been reported with ICI as well as other immune-based therapies. Although key differences between mice and humans will always exist, it is imperative to more critically incorporate these human modifying elements into cancer immunotherapy models in order to more reliably predict clinical outcomes (both positive and negative). This can be done by employing more complex mouse modeling to better mirror potential effects of immune-based interventions.

## Conclusions

Mouse models have been paramount to the discovery of immune checkpoints and the advancement of ICI. Despite these breakthroughs, interpretation of preclinical studies of PD-1/PD-L1 blockade in mice is complicated by several factors. First, and most notably, while the PD-1/PD-L1 pathway has a more defined role in T-cell exhaustion, effects on T-cell priming and other immune cell responses remain largely unknown. Second, there are currently unresolved effects of immune and host-factor differences between young, SPF mice and human cancer patients that can skew interpretation of results. However, moving forward, sex, diet, age, prior infectious challenges, and housing conditions are adjustable variables that, together with the ease and speed of rodent modeling, can be an important investigational tool. While utilization of these modifying conditions can indeed be very costly, they can provide important insights that facilitate translation of the preclinical observations to patients.

## Data Availability

Not applicable.

## Abbreviations

**CAR**: Chimeric antigen receptor
**CMV**: Cytomegalovirus
**CTLA-4**: Cytotoxic T-lymphocyte-associated protein 4
**EBV**: Epstein-Barr Virus
**GEM**: Genetically engineered mouse
**ICIs**: Immune checkpoint inhibitors
**LAG3**: Lymphocyte-activation gene 3
**LCMV**: Lymphocytic choriomeningitis virus
**NK**: Natural killer cells
**OVA**: Ovalbumin
**PD(L)1**: Programmed-cell death (ligand)-1
**SPF**: Specific pathogen-free
**TIGIT**: T-cell immunoreceptor with Ig and ITIM domains
**TIM3**: T-cell immunoglobulin and mucin-domain containing-3

## References

[1] LaFleur MW, Muroyama Y, Drake CG, Sharpe AH (2018). Inhibitors of the PD-1 pathway in tumor therapy. J Immunol.

[2] Schultz Liora, Mackall Crystal (2019). Driving CAR T cell translation forward. Science Translational Medicine.

[3] Wherry EJ, Kurachi M (2015). Molecular and cellular insights into T cell exhaustion. Nat Rev Immunol.

[4] Kamphorst AO, Wieland A, Nasti T, Yang S, Zhang R, Barber DL (2017). Rescue of exhausted CD8 T cells by PD-1-targeted therapies is CD28-dependent. Science.

[5] Barber DL, Wherry EJ, Masopust D, Zhu B, Allison JP, Sharpe AH (2006). Restoring function in exhausted CD8 T cells during chronic viral infection. Nature.

[6] Odorizzi PM, Pauken KE, Paley MA, Sharpe A, Wherry EJ (2015). Genetic absence of PD-1 promotes accumulation of terminally differentiated exhausted CD8+ T cells. J Exp Med.

[7] Mall C, Sckisel GD, Proia DA, Mirsoian A, Grossenbacher SK, Pai CS (2016). Repeated PD-1/PD-L1 monoclonal antibody administration induces fatal xenogeneic hypersensitivity reactions in a murine model of breast cancer. Oncoimmunology.

[8] De La Rochere P, Guil-Luna S, Decaudin D, Azar G, Sidhu SS, Piaggio E (2018). Humanized mice for the study of Immuno-oncology. Trends Immunol.

[9] Nishimura H, Minato N, Nakano T, Honjo T (1998). Immunological studies on PD-1 deficient mice: implication of PD-1 as a negative regulator for B cell responses. Int Immunol.

[10] Lazar-Molnar E, Chen B, Sweeney KA, Wang EJ, Liu W, Lin J (2010). Programmed death-1 (PD-1)-deficient mice are extraordinarily sensitive to tuberculosis. Proc Natl Acad Sci U S A.

[11] Metzger P, Kirchleitner SV, Koenig LM, Horth C, Kobold S, Endres S (2018). Dying cells expose a nuclear antigen cross-reacting with anti-PD-1 monoclonal antibodies. Sci Rep.

[12] Beura Lalit K., Hamilton Sara E., Bi Kevin, Schenkel Jason M., Odumade Oludare A., Casey Kerry A., Thompson Emily A., Fraser Kathryn A., Rosato Pamela C., Filali-Mouhim Ali, Sekaly Rafick P., Jenkins Marc K., Vezys Vaiva, Haining W. Nicholas, Jameson Stephen C., Masopust David (2016). Normalizing the environment recapitulates adult human immune traits in laboratory mice. Nature.

[13] Day Cheryl L., Kaufmann Daniel E., Kiepiela Photini, Brown Julia A., Moodley Eshia S., Reddy Sharon, Mackey Elizabeth W., Miller Joseph D., Leslie Alasdair J., DePierres Chantal, Mncube Zenele, Duraiswamy Jaikumar, Zhu Baogong, Eichbaum Quentin, Altfeld Marcus, Wherry E. John, Coovadia Hoosen M., Goulder Philip J. R., Klenerman Paul, Ahmed Rafi, Freeman Gordon J., Walker Bruce D. (2006). PD-1 expression on HIV-specific T cells is associated with T-cell exhaustion and disease progression. Nature.

[14] McQuade JL, Daniel CR, Hess KR, Mak C, Wang DY, Rai RR (2018). Association of body-mass index and outcomes in patients with metastatic melanoma treated with targeted therapy, immunotherapy, or chemotherapy: a retrospective, multicohort analysis. Lancet Oncol.

[15] Wang Z, Aguilar EG, Luna JI, Dunai C, Khuat LT, Le CT (2019). Paradoxical effects of obesity on T cell function during tumor progression and PD-1 checkpoint blockade. Nat Med.

[16] Kugel CH, Douglass SM, Webster MR, Kaur A, Liu Q, Yin X (2018). Age correlates with response to anti-PD1, reflecting age-related differences in Intratumoral effector and regulatory T-cell populations. Clin Cancer Res.

[17] Routy B, Le Chatelier E, Derosa L, Duong CPM, Alou MT, Daillere R (2018). Gut microbiome influences efficacy of PD-1-based immunotherapy against epithelial tumors. Science..

